# Phenotypic Switching of B16F10 Melanoma Cells as a Stress Adaptation Response to Fe3O4/Salicylic Acid Nanoparticle Therapy

**DOI:** 10.3390/ph14101007

**Published:** 2021-09-30

**Authors:** Ion Mîndrilă, Andrei Osman, Bogdan Mîndrilă, Maria Cristina Predoi, Dan Eduard Mihaiescu, Sandra Alice Buteică

**Affiliations:** 1 Department of Morphology, Faculty of Medicine, University of Medicine and Pharmacy of Craiova, 200349 Craiova, Romania; ion.mindrila@umfcv.ro (I.M.); andrei.osman@umfcv.ro (A.O.); bogdanmindrila01@gmail.com (B.M.); 2 Faculty of Applied Chemistry and Materials Science, University Politehnica of Bucharest, 011061 Bucharest, Romania; danedmih@gmail.com; 3 Faculty of Pharmacy, University of Medicine and Pharmacy of Craiova, 200349 Craiova, Romania; al-ice.buteica@umfcv.ro

**Keywords:** B16F10 melanoma, phenotypic switching, hyperpigmentation, iron oxide nanoparticles, salicylic acid

## Abstract

Melanoma is a melanocyte-derived skin cancer that has a high heterogeneity due to its phenotypic plasticity, a trait that may explain its ability to survive in the case of physical or molecular aggression and to develop resistance to therapy. Therefore, the therapy modulation of phenotypic switching in combination with other treatment modalities could become a common approach in any future therapeutic strategy. In this paper, we used the syngeneic model of B16F10 melanoma implanted in C57BL/6 mice to evaluate the phenotypic changes in melanoma induced by therapy with iron oxide nanoparticles functionalized with salicylic acid (SaIONs). The results of this study showed that the oral administration of the SaIONs aqueous dispersion was followed by phenotypic switching to highly pigmented cells in B16F10 melanoma through a cytotoxicity-induced cell selection mechanism. The hyperpigmentation of melanoma cells by the intra- or extracellular accumulation of melanic pigment deposits was another consequence of the SaIONs therapy. Additional studies are needed to assess the reversibility of SaIONs-induced phenotypic switching and the impact of tumor hyperpigmentation on B16F10 melanoma’s progression and metastasis abilities.

## 1. Introduction

Melanoma is a melanocyte-derived skin cancer with a poor prognosis due to its higher metastatic potential compared to other skin tumors [1], even those from unidentifiable primary locations [2], and the phenotypic plasticity involved in its resistance to therapy [3]. The prognosis is even worse when the melanoma’s primary location is in the mucosal surfaces [4]. Despite a complex therapeutic approach, the management of metastatic melanoma continues to be a major clinical challenge [5], given the rising incidence [6] and increasing mortality [7]. Therapy for melanoma is multimodal, from surgery for the early stages [8] to combinations of chemotherapy, radiation therapy, or immunotherapy for the metastatic stages, and, more recently, to targeted therapy [9,10,11] developed for personalized medicine [5].

The heterogeneity of melanoma cells provides the tumor with the ability to differentiate phenotypic profiles [12,13], each with a specific transcriptional signature, susceptibility to drugs, proliferation rate, and metastatic potential [14]. Melanoma’s phenotypic plasticity allows for the selective evolution of some melanoma cell lines that have adapted to survive in the case of physical or molecular aggression [15]. Therefore, targeting this epigenetically driven phenotypic switching should become a component of future therapeutic strategies to eliminate tumor cells completely [16].

The rapid onset of melanoma’s resistance to therapy, even when applying multimodal and personalized therapy [5], necessitates the development of new therapeutic strategies, such as nanotherapy, which has become more accessible following recent advances in nanomedicine [17]. Nanomedicine is a modern field in melanoma therapy that provides powerful tools for topical therapy [18]; passive and active drug targeting; photothermal, photodynamic, and cytotoxic therapies; and hyperthermia. To date, experimental research has used nanosystems such as inorganic nanoparticles [19,20,21,22], polymeric nanoparticles [23], liposomes [24], carbon nanotubes [25], dendrimers [26], etc.

Their magnetic and toxicological properties, low cost, ease of tailoring in terms of size, morphology, and functionalization enable iron oxide nanoparticles to be widely used as platforms for cancer drug delivery, diagnosis, and targeted multimodal therapy [27,28]. 

In experimental melanoma therapy, functionalized iron oxide nanoparticles are used for drug delivery (either magnetically guided [29] or transdermal [30]), hyperthermia [31,32,33,34], photothermal therapy [35,36], and cytotoxic therapy [37].

SaIONs have proven their therapeutic effect in research models with several types of tumor cells, including melanoma cells [37,38,39], through a synergistic mechanism of apoptosis: mitochondrial-induced stress by the iron oxide core [40] and endoplasmic reticulum-induced stress by the salicylic acid coating [41,42]. Due to the enhanced permeability and retention effects, functionalized iron oxide nanoparticles preferentially accumulate in tumor cells [43]. Iron overloads caused by the exposure of melanoma cells to functionalized iron oxide nanoparticles [44] can interfere with the regulatory mechanisms of melanogenesis and may result in increased pigmentation [45].

Melanin is a vital molecule with many biological functions—as sunscreen and radioprotection [46,47,48], as an antioxidant [49,50], and as a scavenger of toxic materials [51]—and has immunomodulatory [52,53], hepatoprotective [54], anti-inflammatory [55], and anticarcinogenic properties [50]. Melanogenesis has been intensively studied, and the results, sometimes contradictory, have highlighted a wide range of pigmentation effects in modulating melanoma cell traits [56]. The redox status of the melanoma cell is interdependent with melanogenesis [57] and, together with the reactive metal ions in the microenvironment, can determine the pro-oxidant or antioxidant activity of melanin [58]. The melanin pigment has been associated with accelerated melanoma progression [59]; resistance to chemotherapy, radiotherapy, and phototherapy [60]; and/or shortened survival in patients with metastatic melanoma [61].

The pigment-loading process is reversible and the accumulation of pigment granules correlates with the alteration of the nanomechanical properties of the cells and inhibits the migratory properties of melanocytes [55,62]. Moreover, it has been shown that switching between high- and low-pigment production states seems to promote melanoma cell motility [63].

In this work, we performed a morphological and morphometric evaluation of the phenotypic changes and the pigmentation process induced in B16F10 murine melanoma following therapy with an aqueous dispersion of SaIONs. To achieve this, a syngeneic model of B16F10 tissue melanoma implanted in C57BL/6 mice was used, according to a previously reported technique [64]. The phenotypic heterogeneity and tumor pigmentation of the B16F10 murine melanomas were evaluated both in the absence of therapy (M2w and M4w groups sacrificed at two and four weeks, respectively) and after SaIONs therapy, performed by three administration schemes: oral administration (T2w and T4w groups treated for two and four weeks, respectively), intratumoral injection (ITM1w group), and combined administration, first orally, followed by an intratumoral injection (ITO1w group).

## 2. Results

### 2.1. Functionalized Nanoparticles Dispersion Characterization

**Figure 1:**
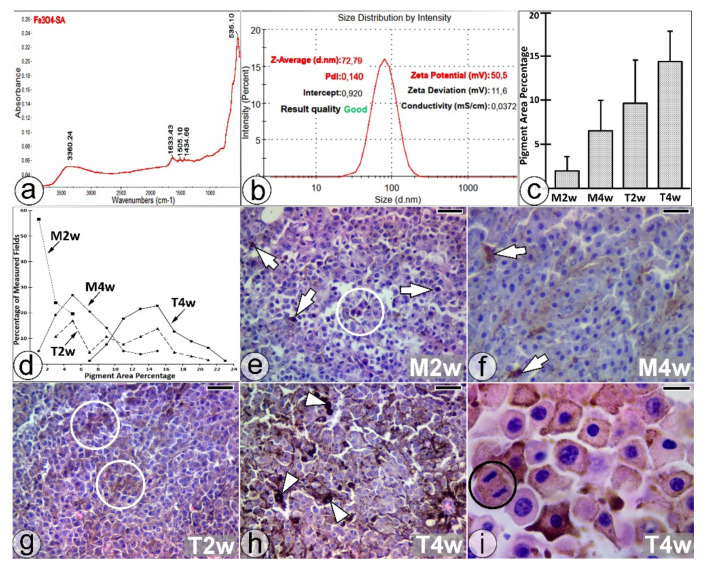
(**a**) FT-IR spectra and (**b**) the DLS size and zeta potential measurements of SaIONs. (**c**) PAP mean values (± SD) and (**d**) percentage ranges of the PAP distribution in the measured fields. (**e**–**i**) Histological sections through untreated (M2w and M4w) and orally treated (T2w and T4w) melanoma tissue, with single PMCs (white arrows), PMC clusters (white circles), HPMCs (white arrow-heads), and PMC mitosis (black circle) highlighted. HE staining. Bar = 25 µm (**e**–**h**); 10 µm (**i**).

The synthesized SaIONs were investigated in order to establish their structure, dimensions, and stability. The presence of the organic layer coating the SaIONs was proven in the FT-IR spectrum by the presence of the characteristic band for the -OH stretching vibrations at 3360 cm^−1^. The C=O stretching band of the carboxyl group can be observed at 1633 cm^−1^, and the characteristic absorption band for the asymmetric stretching vibrations of carboxylate groups can be observed at 1505 cm^−1^. The O-H bending band from the carboxylic group can be observed at 1434 cm^−1^, and the characteristic band assigned to the Fe-O bond vibration can be observed at 535 cm^−1^ (Figure 1a).

DLS analysis (Figure 1b) of the synthesized SaIONs revealed an average hydrodynamic diameter of 73 nm, a good uniformity (a polydispersity index of 0.14), and a very good stability in dispersion (a zeta potential of +50.5 mV). ICP-MS analysis showed a 0.490 mg/mL Fe concentration.

### 2.2. Control Groups Microscopy (M2w and M4w)

All inoculated mice developed tumors, and no deaths were recorded in the studied groups. The areas of programmed tumor necrosis related to tumor size were highlighted by the histological analysis of 2- and 4-week-old B16F10 melanomas harvested from the control groups. For this reason, all areas with tumor necrosis were excluded from the process of measuring the PAP (pigment area percentage) of all the treated or untreated tumors analyzed.

In the M2w tumors with a PAP of 1.97 (±1.6 SD), the distribution of melanin pigment in the measured sections was relatively homogeneous (Figure 1c,d), and the light microscopy revealed a few pigment-laden melanoma cells (PMCs) scattered in the tumor mass, sometimes in small clusters (Figure 1e).

In the M4w tumors, the PAP was 6.5 (±3.4 SD), and the pigment distribution in the measured sections was inhomogeneous (Figure 1c,d), probably due to the growth of PMC clusters in the tumor mass (Figure 1f). A Mann-Whitney U test showed that the PAP of the M4w tumors was significantly higher than that of the M2w tumors, U = 341, z = 7.51, *p* = 0.0001 < 0.05, r = 0.67.

### 2.3. Orally Treated Groups Microscopy (T2w and T4w)

In the group of mice treated with the SaIONs aqueous dispersion administered orally, the average daily intake ranged from 5.21 (±0.83 SD) mL in the first week to 5.16 (± 1.46 SD) mL in the second week, 4.06 (±1.18 SD) mL in the third week, and 4.08 (± 0.85 SD) mL in the fourth week of treatment.

In the T2w tumors, the PAP was 9.6 (±4.9 SD), and the pigment distribution in the measured sections was very inhomogeneous (Figure 1c,d) due to the growth of numerous PMC clusters with different degrees of melanin pigment loading (Figure 1g).

In the T4w tumors, the PAP was 14.3 (±3.4 SD). The melanic pigment distribution pattern in the measured sections was also inhomogeneous (Figure 1c,d), and large, heavily pigmented melanoma cells (HPMCs) began to appear in the PMC clusters (Figure 1h). High-magnification microscopy of the PMC clusters identified pigmented cells in division (Figure 1 i). A Mann–Whitney U test showed that the PAP of the T4w tumors was significantly higher than that of the T2w tumors, U = 1158, z = 5.65, *p* = 0.0001 < 0.05, r = 0.47. In addition, the PAP of the T2w tumors was significantly higher than that of the M2w tumors (U = 97, z = 8.36, p = 0.0001 < 0.05, r = 0.79), and the PAP of the T4w tumors was significantly higher than that of the M4w tumors (U = 375.5, z = 9.49, *p* = 0.0001 < 0.05, r = 0.75).

**Figure 2:**
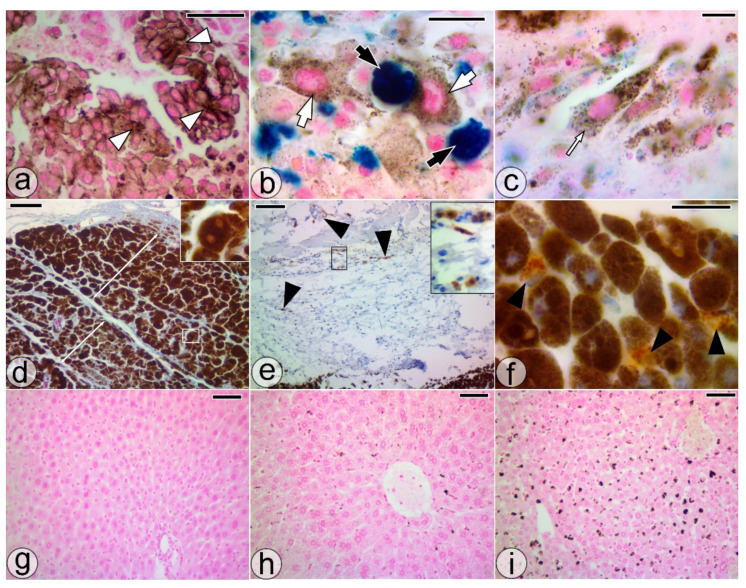
Histological sections through orally treated melanoma (T4w). (**a**) PMC clusters (white arrowheads) with perimembranous accumulation of pigment granules. (**b**,**c**) PMCs (white arrows), NP-PMCs (thin white arrows), and large SaIONs-laden cells (black arrows) in tumor areas with nanoparticle-induced necrosis. (**d**) HPMCs, including some binucleate cells (square), forming overlapping layers at the periphery of the tumor (double-headed arrows). (**e**,**f**) MAC387(+) cells (black arrowheads) in juxtatumoral tissue or spread among HPMCs. (**g**–**i**) Microscopic appearance of the liver with pigment deposits in M4w, T2w, T4w groups, respectively. Perl Prussian blue (**a**–**c**), Azan Trichrome (**d**), and HE staining (**d**,**g**–**i**). Bar = 100 µm (**e**–**i**), 50 µm (**a**,**d**), 10 µm (**b**,**c**,**f**).

An inhomogeneous distribution of melanin granules in the cytoplasm was a trait of the pigment-loading process of most of the melanoma cells (Figure 1i and Figure 2a). PMCs in intimate contact with large SaIONs-laden cells, MAC387(-) pigmented cells with intracytoplasmic granules of SaIONs (NP-PCs), and SaIONs-induced tumor necrosis were highlighted by Perl Prussian blue staining in tumor areas with nanoparticle deposits (Figure 2b,c).

Thick overlapping layers of HPMCs surrounding a core of poorly pigmented melanoma cells has been observed in some treated tumor areas (Figure 2d). In such areas, immunohistochemistry has identified both peritumoral and intratumoral MAC387(+) cells, the latter scattered among HPMCs (Figure 2e,f).

The high production of melanin pigment was also supported by the microscopic analysis of livers harvested from the studied mice. Microscopy of Perls’ Prussian blue-stained liver sections showed more pigment deposits in group T2w and group T4w than in the M4w control group (Figure 2g–i).

### 2.4. Intratumorally Treated Group Microscopy (ITM1w)

**Figure 3:**
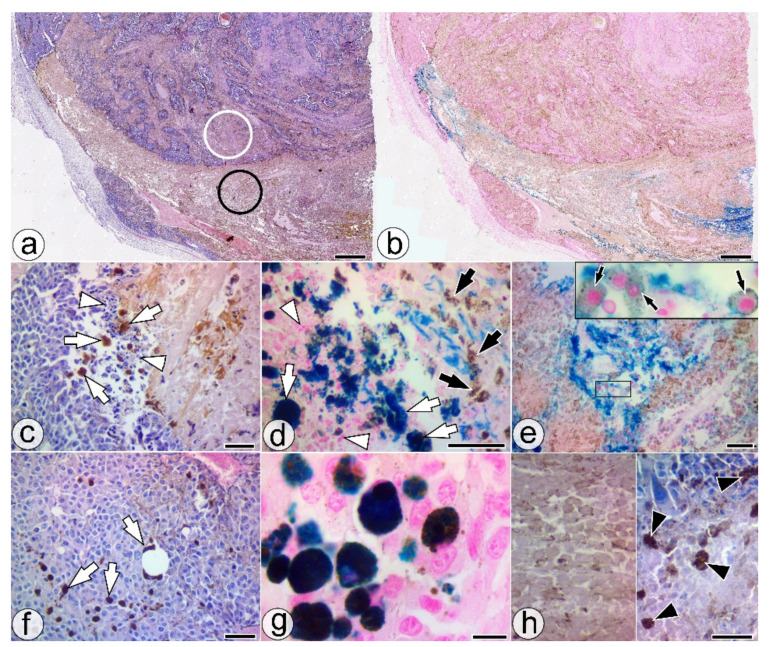
Histological features of the SaIONs injected area in the ITM1w group. (**a**,**b**) Overall appearance of SaIONs-induced necrosis (black circle) in the injected area, with a different morphology from the adjacent programmed necrosis (white circle). (**c**,**d**) The border between the growing tumor and the nanoparticle-induced tumor necrosis with apoptotic bodies (white arrowheads), large MAC387- NP-PCs (white arrows), and melanin-loaded ghost cells without nuclei (black arrows). (**e**) SaIONs-induced tumor necrosis area with nests of viable PMCs (black arrows) surrounded by SaIONs deposits. (**f**,**g**) Melanoma tissue with large migratory NP-PCs (white arrows). (**h**) Higher magnification of the SaIONs-induced necrosis area (left) with pigmented ghost cells without nuclei, and the programmed necrosis area (right) with fragmented nuclei and numerous melanophages (black arrowheads). HE (**a**,**c**,**f**,**h**) and Perls’ Prussian blue staining (**b**,**d**,**e**,**g**). Bar = 10 µm (**g**), 20 µm (**d**,**h**), 50 µm (**c**,**e**,**f**), 1 mm (**a**,**b**).

In light microscopy, the tumor area injected with the SaIONs aqueous dispersion has distinct morphological features compared to neighboring tumor areas with growing tumors or programmed tumor necrosis (Figure 3a,b). Unlike the areas with programmed tumor necrosis, in which numerous pyknotic nuclei, nuclear remains, and an accumulation of large melanophages were present, ghost cells without nuclei, some with well-defined cell outlines that retained their pigment load, could be observed in the SaIONs-induced necrosis (Figure 3h). Atypical tumor cells, exploding cells, apoptotic bodies, pyknotic or fragmented nuclei, and loss of cellular adhesion, highlighted at the border between the SaIONs-injected areas and the growing tumor tissue, can be considered signs of SaIONs’ cytotoxicity on B16F10 melanoma cells (Figure 3c,d). Large NP-PCs formed at the same border could also be identified remotely while migrating in the tumor mass (Figure 3c,d,f,g). These cells appear to be responsible for the removal of SaIONs from the injected area. Nests with viable PMCs have been identified inside the SaIONs-induced necrosis area (Figure 3e).

### 2.5. Orally and Intratumorally Treated Group Microscopy (ITO1w)

**Figure 4:**
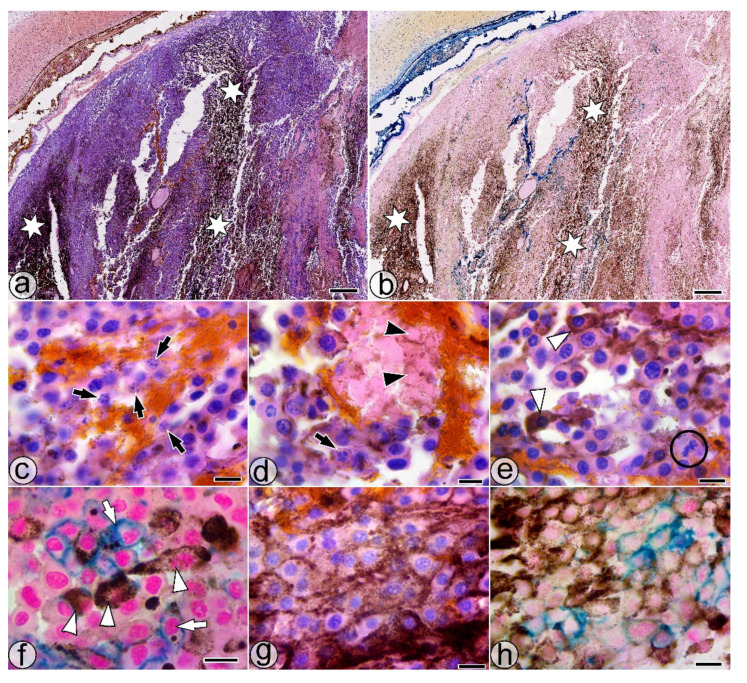
Histological features of the SaIONs-injected area in the ITO1w group. (**a**,**b**) Overall appearance of the hyperpigmentation in the injected area (white stars). (**c**,**d**) Weakly pigmented melanoma cells with fragmented nuclei (black arrows) and ghost cells without nuclei (black arrowheads). (**e**) Area with SaIONs deposits and PMCs with varying degrees of melanin loading (white arrowheads), some in division (black circle). (**f**) Cluster with HPMCs (white arrowheads) and SaIONs-loaded melanoma cells (white arrows). (**g**,**h**) Deposits of pigment and SaIONs in the juxtamembranous regions of the melanoma cells. HE (**a**,**c**,**d**,**e**,**g**) and Perl Prussian blue (**b**,**f**,**h**) staining. Bar = 10 µm (**c**–**h**), 1 mm (**a**,**b**).

The intratumoral injection of the SaIONs aqueous dispersion into the ITO1w group, previously treated orally with the SaIONs aqueous dispersion, resulted in less SaIONs-induced tumor necrosis and a decrease in weakly pigmented melanoma cells, and was particularly accompanied by the hyperpigmentation of the tumor tissue (Figure 4a,b). Pyknotic or fragmented nuclei and ghost cells without nuclei surrounded by SaIONs deposits could be identified mainly in the tumor areas with weakly pigmented melanoma cells (Figure 4c,d).

Signs of SaIONs-induced cytotoxicity were less visible in SaIONs-injected areas associated with tumor hyperpigmentation, where PMCs with progressive degrees of pigment loading and rare PMCs in division may be considered as signs of a tumor’s progressive growth (Figure 4e,f). Findings such as abundant HPMCs and PMCs, large pigment melanosomes in SaIONs-loaded melanoma cells, or the excessive accumulation of pigment granules around the melanoma cells’ membranes in areas with nanoparticle deposits are multiple microscopic aspects of the hyperpigmented tumor that may support the association of injected SaIONs with increased melanogenesis (Figure 4e–h).

## 3. Discussion

The histological examination of the M2w tumors revealed distinct regions of necrosis surrounded by viable regions with progressive tumor growth. This inherent necrosis in B16F10 melanoma tumors has been described even in younger tumors [65] and could be determined by adaptive tumor mechanisms of programmed cell death, which are responsible for the occurrence of so-called programmed necrosis [66,67]. The requirement to remove these areas of necrosis from the study led us to use microscopic morphometry to estimate the degree of melanic pigment loading in the melanoma cells.

Microscopic morphometry of the untreated B16F10 melanoma tumors showed that, in the early stages of tumor growth, pigment-loaded melanoma cells have a relatively homogeneous distribution within the tumor mass. This condition changes in four-week-old tumors, where cells with varying degrees of pigment loading acquire a focal distribution. The results of our study showed that the oral administration of the SaIONs aqueous dispersion was accompanied by an obvious increase in pigment accumulation in the tumor mass, and that the pigment was distributed by PMCs, HPMCs, or extracellular deposits. Usually, the administration of functionalized iron oxide nanoparticles can lead to the iron loading of the tumor [44] by the passive enhanced permeability and retention effect [68]. In human retinal pigment epithelial cells, iron has been shown to increase pigmentation by upregulating the expression of genes involved in melanogenesis [45]. On the other hand, previously reported observations have shown the ability of iron to induce cellular oxidative stress through multiple iron-dependent ROS activation mechanisms: apoptosis and necroptosis [69,70], ferroptosis [71], or pyroptosis [72]. The increased ROS levels in melanoma cells stimulate melanogenesis [73,74,75], an important process that can protect melanoma cells, especially through the free radical scavenging properties [49,50,51] and Fe (II) [76] or Fe (III) [77] chelating abilities of melanin. Moreover, the ability of melanin to bind Fe (III) ions could be involved in the similar perimembranous arrangement of the SaIONs and melanin deposits, highlighted by microscopy in the Perl Prussian blue-stained samples from the ITO1w group (Figure 4h).

Both of the structural components of SaIONs can induce tumor cell stress by altering the activity of intracellular organs: the iron oxide cores alter the function of the mitochondria [40], while the salicylic acid shells alter the function of the endoplasmic reticulum [41,42]. In another study, some of us highlighted the cytotoxic effect of salicylic acid/iron oxide nanoparticles on melanoma B16F10 xenografts [64].

In this study, the SaIONs-induced cytotoxic effect on pigmented or unpigmented melanoma cells was highlighted by microscopy in the SaIONs-injected areas of the previously untreated B16F10 melanoma (ITM1w group). The rapid nuclear dissolution and lack of melanophages in the SaIONs-induced tumor necrosis were specific features. Melanophages had a constant presence in the areas with programmed necrosis. In contrast, migratory NP-PCs, formed at the outer limit of the SaIONs-induced necrosis area, were identified in the neighboring growing tumor. Immunohistochemistry has failed to elucidate the melanocytic or macrophage origin of these cells. MAC387(+) cells were identified mainly peritumorally and less intratumorally, and in the latter case, most of them were scattered among HPMCs (Figure 2e,f).

The microscopic signs of SaIONs-induced cytotoxicity were less visible in the orally treated groups (T2w and T4w), particularly in tumor areas with poorly pigmented melanoma cells. In these tumors, the passive and progressive accumulation of SaIONs favored the appearance of PMCs, HPMCs, and focal accumulations of pigment. Phenotypic heterogeneity and plasticity are traits by which melanoma tumors may adapt to environmental conditions or develop a resistance to treatment [15,16,78]. In fact, these features argued for the use of homogenized syngeneic tumor tissue in this study to achieve the murine model of melanoma B16F10. The phenotype switching of the melanoma cells observed in the tumors of the orally treated groups can be considered an adaptation mechanism of melanoma tumors due to the SaIONs-induced aggression. This mechanism of adaptation may enlighten us as to why the intratumoral injection of SaIONs into previously orally treated tumors (ITM1w group) has resulted in more hyperpigmentation and less cytotoxicity. Cell quiescence [79] was observed in the melanoma B16F10 cells after melanogenesis stimulation [80], and, in this case, it could be considered a reason for the low frequency of mitosis observed in the hyperpigmented tumor areas.

## 4. Materials and Methods

### 4.1. Functionalized Nanoparticles Dispersion

The Sa-IONs were synthesized using pure reagents (KOH, FeCl_3_, FeSO_4_, salicylic acid—Sigma Aldrich) and ultrapure water (from a Millipore Elix 5 water purifier) by a modified Massart synthesis, as reported earlier [81]. Briefly, by using an ultrasound bath, 4 grams of FeCl_3_ and 2.5 grams of FeSO_4_ (stoichiometric ratios of Fe II and Fe III) dissolved in 500 mL of ultrapure water were precipitated under basic conditions (20 g KOH, 1 g salicylic acid, and 500 mL ultrapure water) at 50 °C. The excess KOH and salicylate were removed by repeated washings with ultrapure water, sonication, and magnetic separation.

The synthesized SA-IONs were characterized by FT-IR, DLS, and ICP-MS analyses. An FT-IR analysis was used to evaluate the structure of the Fe3O4/Salicylic acid core–shell nanoparticles. The FT-IR spectra were recorded using a Zn-Se window H-ATR mounted on a Thermo Nicolet 6700 spectrometer. A particle-size distribution analysis of the synthesized nanoparticles was performed by DLS with a Zetasizer Nano ZS (Malvern Instruments Ltd., United Kingdom). The mean diameter and the polydispersity index of the nanoparticles in the dispersion were measured at a spreading angle of 90° and a temperature of 25 °C. All the measured data were given as the average of three measurements. An ICP-MS analysis was used to determine the content of iron in suspended Fe_3_O_4_ nanoparticles. Prior to the ICP-MS analysis (Agilent 8800, Agilent Technologies), the sample was diluted 10 times with ultrapure water. A calibration curve, ranging from 1 ppb to 100 ppb, was prepared using a multielement standard solution (multi-element calibration standard-2A, Agilent Technologies). Linear calibrations with a correlation coefficient greater than 0.99 were obtained for all elements.

### 4.2. Animals

Eighteen eight-week-old female C57BL/6 mice weighing between 17 and 20 grams obtained from the University of Medicine and Pharmacy of Craiova Animal Facility were used in this experiment. They were kept in stainless steel cages and had free access to water and standard food. A room temperature of 21 °C and a light/dark cycle of 12 hours were set. The protocol of the experiment was approved by the Committee of Ethics and Scientific Deontology of the University of Medicine and Pharmacy of Craiova (139/20.12.2019).

A four-week-old B16F10 cell melanoma, developed in a C57BL/6 female mouse that was part of the control group in another experiment, was used as a source of tumor tissue (B16F10 cells were obtained from The Institute of Biology of the Romanian Academy). Under sterile conditions, 2.5 mL of melanoma tissue were aspirated with an 18 G needle assembled onto a 10 mL syringe from the Sevorane®-anesthetized donor mouse. 5 mL of saline were added to the collected tissue, and the syringe was shaken manually to homogenize the contents. All maneuvers applied to the mice in this experiment (subcutaneous injection of tumor homogenate, intratumoral injection of nanoparticle dispersion, and sacrificing of mice) were performed under Sevorane® anesthesia.

Each mouse received a quantity of 0.2 mL of a homogenate of melanoma tumor tissue by subcutaneous injection into a previously shaved area of the right flank. Next, the mice were randomly divided into 2 groups (*n* = 9).

#### 4.2.1. Group 1

Two weeks after the subcutaneous implantation of the tumor tissue homogenate, three mice randomly selected from this group were injected intratumorally with 0.5 mL of the SaIONs aqueous dispersion, and another three mice were sacrificed (M2w group). The remaining three mice were sacrificed after four weeks of tumor development (M4w group). The mice that were injected intratumorally with the aqueous dispersion of nanoparticles were sacrificed one week after the treatment (ITM1w group).

#### 4.2.2. Group 2

These mice had free access throughout the experiment to the SaIONs aqueous dispersion, which replaced their drinking water 48 hours after the subcutaneous implantation of the tumor homogenate. After two weeks of oral administration of the SaIONs aqueous dispersion, three randomly selected mice were injected intratumorally with 0.5 mL of the SaIONs aqueous dispersion, and another three mice were sacrificed (T2w group). The remaining three mice were sacrificed after four weeks of oral administration of the SaIONs aqueous dispersion (T4w group). The mice injected intratumorally with the SaIONs aqueous dispersion were sacrificed one week after the treatment (ITO1w group).

All the sacrificed mice were autopsied to assess tumor morphology and to harvest primary tumors and viscera for histological and immunohistochemical evaluation.

### 4.3. Histopathology

The tissue fragments were processed for fixation in neutral buffered formalin (4%) and paraffin embedding, then slides were cut on a Microm HM355 rotary microtome and collected on poly-L-lysine coated slides.

For conventional light microscopy, the 5 µm-thick sections were stained with H&E, Azan Trichromic, and Perls’ Prussian blue. To estimate the tumor pigmentation, we measured the pigment area percentage (PAP) on Perl Prussian blue-stained sections: PAP = pigment granule area *100/measured area. Therefore, 50 fields taken with the x40 objective were processed for each studied tumor using the Image-Pro Plus image processing and analysis software.

For immunohistochemistry, the slides were deparaffinated and re-hydrated, processed for antigen retrieval by boiling in citrate buffer (0.1M, pH 6). Endogenous peroxidase blocked in 1% water peroxide and unspecific binding sites were blocked with skimmed milk. The primary antibody was incubated on the slides overnight (mouse anti-macrophage, clone MAC387, LSBio, Seattle WA, USA, 1:10) at 4 °C, and the next day the signal was amplified with an anti-mouse alkaline phosphatase-labelled polymer (Histofine Simple Stain™ AP, Nichirei Biosciences Inc., Tokyo, Japan) for 1 hour. Then, the signal was visualized with 3,3′-Diaminobenzidine (Nichirei Biosciences Inc.), and the sections were counterstained with hematoxylin and coverslipped using a xylene-based mounting medium (Sigma).

### 4.4. Statistical Analysis

The results are presented as mean ± standard deviation (SD). The studied groups were compared two by two using the Mann–Whitney U test, having set the significance level at 0.05. Data were analyzed using SPSS version 16 (SPSS, Inc., Chicago, IL, USA).

## 5. Conclusions

The results of this study showed that SaIONs therapy can be used in the phenotypic modulation of B16F10 melanoma. The direct intratumoral administration of high doses of SaIONs was followed by tumor necrosis, induced by the cytotoxic effect of SaIONs on melanoma cells. Therapy with low and repeated doses of orally administered SaIONs induced a phenotypic switching in the melanoma, favoring the appearance of pigment-laden melanoma cells through a cytotoxicity-induced cell selection mechanism. Direct intratumoral administration of high doses of SaIONs in previously phenotypically switched tumors induced more hyperpigmentation and less cytotoxicity in the treated B16F10 melanoma. Additional studies are needed to assess the reversibility of SaIONs-induced phenotypic switching and the impact of tumor hyperpigmentation on B16F10 melanoma’s progression and metastasis abilities.

## Data Availability

All data has been present in main text.
